# Tumour mutational burden using a targeted panel approach for comprehensive tumour profiling focusing on colorectal cancer

**DOI:** 10.1186/s13053-025-00308-9

**Published:** 2025-02-28

**Authors:** Rodney J. Scott, Andrew Ziolkowski, David Mossman, Michael Hipwell

**Affiliations:** 1 Division of Molecular Medicine, NSW Health Pathology, New Lambton, NSW 2305 Australia; 2 Hunter Medical Research Institute, The School of Biomedical Sciences, Faculty of Health and Wellbeing, University of Newcastle, Callaghan, NSW 2308 Australia

**Keywords:** Tumour Mutational Burden, Applications, Colorectal cancer, Epigenetic change and large deletions

## Abstract

There is an increasing recognition that comprehensive tumour profiling (CTP) represents an important adjunct to the diagnosis of malignancy providing not only an assessment of how many mutations there are in any given tumour which reflects the probability of immune checkpoint inhibitor success, but also which mutations are associated with targeted therapies, a signature that reflects environmental insult and potentially the identification of cancers of unknown origin.

This short review describes an approach to assaying tumour mutational burden (TMB), what the difficulties are in the assessment of the TMB and what it can be applied to in regards to improving patient outcomes. A final section of the review delves into some examples of colorectal cancer studies that identify findings that suggest there remains much to learn about tumour development.

## Introduction

Why tumour mutational burden in a symposium that focuses on familial aspects of human cancer? The answer to this question is based on the reduction of DNA sequencing costs that has and continues to decline, allowing more individuals to benefit from knowledge about the molecular events that underpin their cancer development. This short review will concentrate on what is entailed in assessing tumour mutational burden and why this is an important adjunct to cancer patient care.

Why assess tumour mutational burden (TMB) is an often asked question that can be answered at various levels. The molecular profile of a tumour can aid in the diagnosis and treatment of disease, especially in those tumours that are difficult to classify using more traditional approaches to histopathological diagnosis. With respect to TMB, a major significant driver is that the higher the TMB score, the more likely immune checkpoint inhibitors (ICIs), such as PD-1, PD-L1, CTLA-4 and LAG-3 blockers, will be effective in treating the disease (for review see Choucair et al. 2020 [1]). This is the primary goal of undertaking this type of analysis but there are other benefits as well that include the identification of molecular targets that are likely to result in favorable response to particular therapies (additional to ICIs), the confirmation of a histopathological diagnosis, the assessment of microsatellite instability, the identification of a mutational signature associated with a particular environmental exposure and the potential diagnosis of the tissue of origin of cancers of unknown primary disease. In addition, by screening multiple genes in a single reaction, knowledge about the causes of disease will be forthcoming that include the role of inherited predispositions to cancer that may have remained hidden due to the absence of any remarkable family history of disease.

### Approaches to determining tumour mutation burden

The TMB, which is usually reported as the number of nonsynonymous single nucleotide variants per megabase, can be assessed using a variety of different techniques, all of which require massively parallel sequencing to identify the number of mutations across the genome. Clearly, whole genome sequencing will provide the most comprehensive catalogue of mutational changes within the tumour genome and second to this is whole exome sequencing, even though it only covers ~ 2% of the genome. Both these methods provide relatively good concordance and would be considered the preferred approach but the costs embrace not only monetary costs but also data interpretation and storage costs, which are prohibitive, even with the ever reducing expenditure in sequencing. An alternative is to use a large targeted panel that is large enough to determine the average mutational burden across the genome yet not so demanding with respect to data analysis and actual sequencing costs. For the remainder of this mini-review only the panel approach will be discussed.

Most targeted panel sequencing assays that are capable of estimating TMB with sufficient confidence utilise a panel of greater than 300 genes that includes both an investigation of genomic DNA as well as a limited RNA fusion panel to cover fusions commonly observed in solid tumours that are amenable to specific therapeutic options. Many of the genes utilised by a TMB assay are relevant to hereditary cancer and include all the usual suspects (i.e. *BRCA1*,* BRCA2*,* APC*,* MSH2*,* MLH1*,* MSH6*,* PMS2* etc.). Even with this approach there are substantial interpretation challenges in providing a logical and meaningful report from each tumour studied. The optimal number of genes in the panel is ~ 500, most of which are well known cancer associated genes that are distributed across the genome as evenly as possible to provide a best estimate of mutations per Mb of DNA. The higher the number of somatic variants identified per Mb the greater the probability that the tumour will respond to immune checkpoint inhibitors, however PD-L1 expression on tumour cells remains a key independent biomarker that will strongly influence the response to ICI and as such both TMB and PD-L1 biomarkers should be considered independently to access the potential benefit of ICI therapy [2].

With the genome being 3.0 Gb in size and at one mutation per Mb it is to be expected that there would be ~ 3,000 somatic variants present if there was a mutation every Mb. The common consensus for TMB-high is 10 mutations per megabase, suggesting there would be ~ 30,000 somatic variants detected if the entire genome is studied. Since only 1.94 Mb is used to identify the average mutation rate per Mb, it would be expected that at most two mutations would be detected if one occurred every Mb. If the TMB was ten times higher, we would expect, at most, to analyse 20 variants. The analysis of TMB must take into account the presence of inherited differences (these would include recognised polymorphisms) and remove these, otherwise there would be an unacceptably high TMB score since polymorphisms occur at a rate of ~ 1/Kb. This makes the assessment of TMB complex and not something that is straight forward. As a result of these complexities an attention to detail is required to ensure that only somatic variants (i.e., those changes acquired by the tumour) are assessed and then only those that have a functional consequence are reported.

### What is the targeted panel approach for comprehensive tumour profiling used for

Apart from providing a TMB score this type of assay provides information on the level of microsatellite instability, the spectrum of mutations present within a tumour, specific biomarker information for the selection of targeted therapies, the detection of copy number variants at the gene level and the detection of key fusion genes associated with solid tumours. Due to the assay only employing ~ 500 genes, the relative accuracy of chromosomal copy number variation is limited and as such only estimates can be provided based on one or more gene deletion or duplication calls.

Often, but not always, a high TMB is associated with an MSI high score. The higher the TMB score, the more likely the production of neoantigens, which in conjunction with frameshift peptides (due to microsatellite instability) results in a tumour that is highly immunogenic and very susceptible to immune checkpoint inhibitors [3]. Intriguingly, not all TMB high tumours are MSI high, nor are all MSI tumours always TMB high [4]. This conundrum is difficult to reconcile but appears to be related to the tissue of origin. For gastrointestinal cancers that include stomach, duodenum and small intestinal adenocarcinomas co-existence of MSI with a high TMB occurred in those samples that were TMB high whereas in other tumour types, such as melanoma, squamous cell carcinoma and lung carcinoma it is very rarely observed to co-occur [4].

Some tumours with high TMBs can be linked to specific environmental exposures, such as seen in melanomas that are a result of UV-light exposure. There has been considerable work undertaken by various groups internationally to determine if there are specific mutational spectra and as such several signatures have been identified but many remain to be characterised (see https://cancer.sanger.ac.uk/signatures/sbs/).

### An overview of annotation

Given that screening over 500 genes, most of which have been associated with cancer in one form or another can result in hundreds if not thousands for somatic variants, many of which are novel, special consideration needs to be given in interpreting whether these changes are implicated in disease or not. Prior to a report being released a considerable amount of curation is undertaken that removes many benign changes and assigns pathogenicity to those that fulfil a complex set of guidelines as determined by following a standard operating procedure that promotes reproducibility between different laboratories [5]. These consensus guidelines were first devised by The Association for Molecular Pathology (AMP), American Society of Clinical Oncology (ASCO) and the College of American Pathologists (CAP) to remove as much discrepancy in interpretation as possible [6]. The classification of somatic variants that takes into consideration population data, functional and predictive data as well as cancer hotspots (i.e. how many times a specific variant has been reported) and computational evidence. Together these lines of evidence provide a reliable interpretation of most variants that are identified by this assay and are now recommended by the Clinical Genomics Resource (ClinGen), the Cancer Genomics Consortium (CGC), and the Variant Interpretation for Cancer Consortium (VICC) [6].

### Beyond TMB in comprehensive tumour profiling: a focus on colorectal cancer

Apart from TMB and MSI, an understanding of the mutational landscape can be valuable in determining prognostication, therapeutic options, directing which are the most efficacious approaches to treating colorectal cancer and likely site of origin. For example, the identification of APC mutations invariably indicates a colorectal tumour; TP53 mutations generally confer a poor prognosis for most tumour types. Mutations in genes that are associated with a targeted therapy are readily identified, these include tyrosine kinases (such as EGFR mutations that suggest response to tyrosine kinase inhibitors; and NTRK inhibitors that are likely to be effective in lung cancer patients) [7, 8] and tumours that are expected to respond to Poly (ADP ribose) polymerase (PARP) inhibitor treatment (for review see Slade [9]). There are a number of other targets that can be used to direct therapy all of which may be of value in the treatment of cancers that develop in people who carry a genetic predisposition to disease. This is especially interesting with respect to MSI high tumours that respond well to immune check point inhibitors [10]. New classes of treatment are beginning to appear that are specific for Lynch syndrome that enhance immune surveillance against frameshift peptides [10], the presence of which is indicative of a high TMB driven by MSI.

The use of large panels to identify molecular features of specific tumour types can yield surprising results as well as those that would be expected. For example, in rare colorectal diseases such as medullary carcinoma of the colon, Jabbal et al. [11] described the entity as being poorly differentiated, a tendency to be right sided and having a high prevalence of MSI. The results of the analysis revealed a high TMB and MSI. Intriguingly, neither APC mutations or DNA mismatch repair (MMR) mutations were identified in MLH1, MSH2, MSH6 or PMS2. Since MSI was clearly evident and no MMR mutations were detected by panel-base comprehensive tumour profiling it is likely, but not proven, that either epigenetic modification resulting in the loss of expression of one of the MMR genes (most likely MLH1) has occurred or there is deletion not identified (for example between EPCAM and MSH2) that results in the silencing of MSH2 [12].

*APC* mutations invariably indicate a tumour of colorectal origin but can occur in association with *MSH6*, which predicts DNA microsatellite instability [13] but does not display such a feature. A plausible explanation comes from the recognition that MSH6 appears to be linked to a higher number of single nucleotide variants (SNVs) and a reduced frequency of insertion/deletion mutations (INDELs). This is most likely due to the MSH2-MSH6 complex being engaged in the repair of SNVs and almost redundant for INDEL repair [14]. Comprehensive tumour profiling also enables the assessment of gene amplifications and limited yet well-characterised potentially actionable RNA fusions. For example, HER2 overexpression is present in up to 5% of metastatic colorectal cancers and is a well-established negative predictive biomarker of anti-EGFR-targeted therapy [15]. Gene fusions are rare in colorectal cancer, however, identifying additional candidates for target therapy by identifying gene fusions including NTRK, FGFR and RET has clear clinical benefits. Detecting the above additional biomarkers by CTP is much more efficient and comprehensive than relying on a small panel supplementary investigation such as RT-qPCR, CISH or FISH (for example, see Sforza et al. [16] and Zhang et al. [17]).

## Data Availability

No datasets were generated or analysed during the current study.
